# Synergistic variation of rhizosphere soil phosphorus availability and microbial diversity with stand age in plantations of the endangered tree species *Parashorea chinensis*

**DOI:** 10.3389/fpls.2024.1372634

**Published:** 2024-04-12

**Authors:** Wannian Li, Saif Ullah, Fang Liu, Fuchun Deng, Xiaomei Han, Songdian Huang, Yuanyuan Xu, Mei Yang

**Affiliations:** ^1^ Guangxi Colleges and Universities Key Laboratory for Cultivation and Utilization of Subtropical Forest Plantation, Guangxi University, Nanning, China; ^2^ Nanning Arboretum, Guangxi Zhuang Autonomous Region, Nanning, China; ^3^ Guangxi Key Laboratory of Forest Ecology and Conservation, College of Forestry, Guangxi University, Nanning, China

**Keywords:** acidic soil, endangered species, phosphorus availability, phosphorus fraction transformation, rhizosphere microbial diversity

## Abstract

**Introduction:**

Soil physicochemical properties and nutrient composition play a significant role in shaping microbial communities, and facilitating soil phosphorus (P) transformation. However, studies on the mechanisms of interactions between P transformation characteristics and rhizosphere microbial diversity in P-deficient soils on longer time scales are still limited.

**Methods:**

In this study, rhizosphere soils were collected from a pure plantation of *Parashorea chinensis* (*P. chinensis*) at six stand ages in the subtropical China, and the dynamic transformation characteristics of microbial diversity and P fractions were analyzed to reveal the variation of their interactions with age.

**Results:**

Our findings revealed that the rhizosphere soils across stand ages were in a strongly acidic and P-deficient state, with pH values ranging from 3.4 to 4.6, and available P contents ranging from 2.6 to 7.9 mg·kg^-1^. The adsorption of P by Fe^3+^ and presence of high levels of steady-state organic P highly restricted the availability of P in soil. On long time scales, acid phosphatase activity and microbial biomass P were the main drivers of P activation. Moreover, pH, available P, and ammonium nitrogen were identified as key factors driving microbial community diversity. As stand age increased, most of the nutrient content indicators firstly increased and then decreased, the conversion of other forms of P to bio-available P became difficult, P availability and soil fertility began to decline. However, bacteria were still able to maintain stable species abundance and diversity. In contrast, stand age had a greater effect on the diversity of the fungal community than on the bacteria. The Shannon and Simpson indices varied by 4.81 and 0.70 for the fungi, respectively, compared to only 1.91 and 0.06 for the bacteria. Microorganisms play a dominant role in the development of their relationship with soil P.

**Discussion:**

In conclusion, rhizosphere microorganisms in *P. chinensis* plantations gradually adapt to the acidic, low P environment over time. This adaptation is conducive to maintaining P bioeffectiveness and alleviating P limitation.

## 1 Introduction

Phosphorus (P) is the second most important soil nutrient for plants after nitrogen, and is also an essential life element for energy transport, cell structure and nucleic acid formation in plants (Margalef et al., 2017). Adequate P supply is particularly important for the establishment of plant organs and functions to improve vitality and disease resistance, but when P is deficient it usually limits plant growth (Hedley et al., 1982a; Wang et al., 2020). Most of the phosphate in the soil will bind to the surface of material with a negative charge or combine with clay minerals and oxides, hydroxides and cationic precipitates of Fe^3+^, Al^3+^ and Ca^2+^, becoming ineffective P (Philippe, 2001; Frédéric, 2016; Emsens et al., 2017; Marie et al., 2020).These factors can combine to cause a deficiency of bio-available P in the soil (Menezes-Blackburn et al., 2018). Therefore, it is necessary to conduct studies on soil P availability, which is defined as the potential supply of available P released from the soil through the rhizosphere activity of plant roots and microorganisms (Wu et al., 2019). The contribution of different P forms to soil P effectiveness varies greatly, and the conversion between P forms plays a major role in determining P availability (DucoussoDétrez et al., 2022; Xu et al., 2022). Both organic and insoluble inorganic P can be converted to phosphate ions by microorganisms and then directly absorbed by plants (Timothy et al., 2017). Therefore, the ability of soil microorganisms to dissolve P adequately reflects the rhizosphere process of P transformation and is usually used as a direct indicator of P fertility (Xu et al., 2022). However, most of the current studies on soil P cycling and transformation have focused on non-rhizosphere soils, neglecting the interaction between P forms transformed in rhizosphere soils and their availability with microbial communities.

It has been found that rhizosphere microorganisms are the main drivers of P mineral transformation and P nutrient mobilisation from the earliest stages of soil genesis (Neori et al., 2007). For example, the well-developed mycelial network of mycorrhizal fungi symbiotic with the plant root system helps plants to absorb P from the soil (Gavito et al., 2003; Jiang et al., 2021). Large microbial pool coupling with root acquisitive traits increased crop P uptake (Zhang et al., 2022). Microorganisms drove transformations from organic P to inorganic P (Shi et al., 2023). The rhizosphere microorganisms accelerate the release of unstable P by releasing protons, carboxylates and secreting acid phosphatase to increase orthophosphate and enhance the content and mobility of available P in the soil (Chen et al., 2018; Marie, 2020). It is evident that rhizosphere soil microorganisms play an important role in improving the efficiency and availability of conversion between P fractions. Therefore, it is essential to link soil P chemistry with soil microbiology as a means of understanding changes in microbial community composition in relation to P availability (DucoussoDétrez et al., 2022). In addition, the effect of stand age should be taken into account. The relationship between plant rhizosphere soil microorganisms and P transformation changes continuously over time, and the balance between plant P uptake and soil P supply, as well as the spatial distribution pattern of P are affected by stand age (Liu et al., 2015, 2018). In addition to the effects of forest age, changes in land use practices and tree species grown also profoundly affect soil energy flow and nutrient cycling. Compared with natural forests, artificial management usually has negative impacts on forest soil matter cycling and microbial activities (Liao et al., 2012). In the subtropical mountains of southern China, afforestation causes changes in the amount of apoplastic material and soil nutrient status, which drive indirect variations in soil microbial diversity (Yu et al., 2012). The plantation management activities of non-native tree species may change the physical, chemical and biological properties of soil and reduce soil quality (Zarafshar et al., 2020). Therefore, it appears to be of great interest to explore the synergistic changes in soil nutrient effectiveness and microorganisms after ex situ conservation of endangered tree species.

*Parashorea chinensis* Wang Hsie (*P. chinensis*) is a tall tree of Dipterocarpaceae, which plays a unique ecological function as a dominant species in Chinese tropical rainforests and has irreplaceable research and economic value. It is a national first-class protected wild plant endemic to China, and IUCN has listed it as an endangered tree species (Tan et al., 2017). Currently, 10 of the 13 species of Dipterocarpaceae trees native to China have suffered serious damage to their resources and are listed on the national first or second level of protection (Duan et al., 2019). The species of this family are also seriously threatened and endangered in many Southeast Asian countries, and most of them are in a protected status (Curran et al., 1999; Curran and Webb, 2000; Kettle, 2010). Currently, the natural habitats and plantation soils of *P. chinensis* in China are facing a scarcity of available P (Li W, et al., 2020). P limitation is also a widespread problem in tropical and subtropical forest soil in China, and it has become a major challenge in restoring the population size of Dipterocarpaceae species, severely limiting stand fertility and productivity (Laliberte et al., 2014; Yu et al., 2022). Therefore, the this study aims to investigate soil P bioavailability and microbial community changes and interactions in plantation stands within a subtropical red soil region. The main objective was to clarify the primary physicochemical factors driving these changes, providing a scientific basis for alleviating P limitation of endangered species in acidic P-deficient areas and improving soil fertility management. The hypotheses proposed are as (1) soil nutrient content and P availability decrease with stand age, and (2) soil microorganisms gradually adapt to the rhizosphere environment with increasing stand age, thereby positively contributing to the improvement of soil P conversion efficiency and P availability.

## 2 Materials and methods

### 2.1 Experimental site

In order to save the endangered species *P. chinensis*, its plantations were established in different years in the camping area (22°37′57″N, 108°18′47″E) in the Nanning Arboretum of the Guangxi Zhuang Autonomous Region from 1978 onwards. All stands are located south of the Tropic of Cancer, the zonal vegetation is dominated by typical subtropical monsoonal evergreen broadleaf forests, and the soil type is latosol. Due to the highly irregular seed bearing time of *P. chinensis*, some years do not even produce seeds for cultivation. In addition, the seeds germinate very quickly once matured making it impossible to store them for long periods of time, and those that do germinate have a very low viability rate. These factors make it difficult in some years to collect enough seeds for artificial seedlings and large-scale afforestation, so the selection of stands for this study was very limited, resulting in a heterogeneous age gradient in these stands (**Table 1**). The initial planting density was 400 plants·ha^-1^ when *P. chinensis* was first introduced in 1978 and approximately 1,665 plants·ha^-1^ in other stands. Starting from 2011, a base fertilizer was applied before planting and a follow-up dose was applied in May of each of the first two years after planting, after which no more fertilizer was applied. The amount of fertilizer applied was 0.25 kg·plant^-1^ and the mass ratio of active ingredients was N:P:K = 15:6:9, and the undergrowth was cut down and weeded to promote seedling growth.

**Table 1:** Coordinates and topographic information of the sample points.

Stand	Year of afforestation	Stand age (year)	East longitude	Northern latitude	Altitude (m)	Position of slope	Slope	Extant plant line distance
A1	2019	1	108°17’56.52”	22°37’21.53”	144.8	Downside	15~25°	2 m×3 m
A2	2018	2	108°17’4.03”	22°37’25.38”	151.9	Middle	15~25°	2 m×3 m
A3	2017	3	108°17’0.88”	22°37’25.19”	144.1	Middle	10~25°	2 m×3 m
A8	2012	8	108°18’57.52”	22°37’21.92”	137.5	Downside	10~20°	2 m×3 m
A9	2011	9	108°18’39.81”	22°37’55.50”	140.7	Downside	10~20°	2 m×3 m
A42	1978	42	108°17’11.37”	22°43’41.95”	120.4	Downside	10~15°	5 m×5 m

NW, northwest; NE, northeast; SW, southwest; E, east.

### 2.2 Experimental design and soil sampling

We set up sample plots in July 2020 within pure plantation stands of *P. chinensis* of six different forest ages. These stands are scattered in adjacent camping areas, so that the altitude, climate, topography and initial soil physicochemical properties are similar (**Tables 1**, **2**). This prevents distortion due to soil spatial variations. The commonly adopted method is the ‘space-for-time’ approach. Specifically, with dispersed sites of different ages exhibiting similar initial status, synchronous-sampling at these sites is identical to resampling at the same site at various ages. We set up three standard sample plots of 20 m × 20 m (400 m^2^) under each stand age, and the spacing of the standard sample plots was set over 800 m to prevent pseudo-replication and reduce spatial self-correlation (Tang and Wang, 2022).

**Table 2:** Initial values of soil nutrient content before afforestation in 2011.

Soil depth	pH	Total Nitrogen, TN (g·kg^−1^)	Total Phosphorus, TP (g·kg^−1^)	Total Potassium, TK (g·kg^−1^)	Ammonium Nitrogen, AN (mg·kg^−1^)	Nitrate Nitrogen, NN (mg·kg^−1^)	Available Phosphorus, AP (mg·kg^−1^)	Available Potassium, AK (mg·kg^−1^)
0~20 cm	3.74	1.84	0.26	3.98	7.26	4.58	2.56	55.24
20~40 cm	3.43	1.35	0.23	7.02	3.28	3.90	3.20	50.59

Five standard trees representing the average growth level of the stand were selected in each standard sample plot of each stand age, and their rhizosphere soil samples were collected and divided into three parts after thorough mixing. The first part was air-dried, ground and sieved (sieve 0.15 mm) for the determination of soil physicochemical properties and nutrient content; the second part was stored in a refrigerator at 4°C in fresh condition for the determination of soil enzyme activity and microbial biomass P; the third part was transported immediately after collection in liquid nitrogen and stored in an ultra-low temperature refrigerator (-80°C) in the laboratory for high-throughput sequencing of soil bacteria and fungi. Samples were collected by avoiding fertilization points at the root base of trees under the guidance of campers, and then removing the top layer of soil about 5 cm thick with a sterilized shovel. The fine roots were then excavated at a depth range of 40 cm, and the soil attached to the fine roots was collected by shaking and brushing as rhizosphere soil samples (Clausing et al., 2021).

### 2.3 Analysis of soil physicochemical properties and the determination of nutrient content

The soil was air dried and sieved (0.15 mm mesh), and the soil pH was measured using a pH meter (model PHBJ-260, Lei-ci, Shanghai, China). Soil organic matter (SOM) content was analyzed by the potassium dichromate volumetric method. Soil total nitrogen, phosphorus, and potassium (TN, TP, TK) and available nitrogen (AN, NN), available phosphorus and potassium (AP, AK) contents were analyzed by Kjeldahl, Molybdenum Antimony Anti-Colorimetric Method, and Flame spectrophotometry methods, respectively (Bao, 2000; Kammann et al., 2015).

### 2.4 Determination of phosphotrophic properties in rhizosphere

Soil microbial biomass phosphorus (SMBP) was measured by chloroform fumigation using fresh soil. $SMBP=E_{pt}/K_{p}$ , *E_pt_* denotes the difference between the amount of organic P measured in fumigated soil and in unfumigated soil, and the $K_{p}$ denotes the conversion coefficient, a value of 0.4 (Hedley et al., 1982b).

Phosphorus activation coefficient (PAC) is the percentage of AP (mg·kg^-1^) to TP (g·kg^-1^) (Chen et al., 2020). SMBP/TP is considered as an indicator of P utilization efficiency by microorganisms (Li Q, et al., 2020). Activity of acid phosphatase (ACP) was determined by p-Nitrophenylphosphoric acid method using an ultraviolet spectrophotometer (UV-1900i, Shimadzu Corporation, Japan). PAC could measure the effectiveness of soil P; when PAC > 2.0%, it indicates that TP is easily converted to AP, and when PAC< 2.0%, it indicates the opposite (Yang et al., 2010).

The specific classification of inorganic and organic P follows the method mentioned by He et al (He et al., 2021). shows in **Table 3**. The content of the various inorganic and organic P fractions was determined according to the Chang-Jackson method (Chang and Jackson, 1957) and the Bowman-Cole method (Bowman and Cole, 1978), respectively.

**Table 3:** The relationship between phosphorus component analysis.

	Phosphorus major forms	Phosphorus fractions
Total phosphorus, TP	Organic phosphorus, Org P	Labile organic phosphorus, LOP
		Moderately labile organic phosphorus, MLOP
		Moderately resistant organic phosphorus, MROP
		Highly resistant organic phosphorus, HROP
	Inorganic phosphorus, Ino P	Aluminum-bound phosphorus, Al-P
		Iron-bound phosphorus, Fe-P
		Calcium-bound phosphorus, Ca-P
		Insoluble phosphorus, O-P
	Unextracted phosphorus, UP	

### 2.5 High-throughput sequencing analysis of fungi and bacteria

The rhizosphere soil samples of this study were determined for high-throughput sequencing by Beijing Nuohe Zhiyuan Technology Co., Ltd. for genomic DNA extraction by CTAB (Cetyltrimethylammonium Bromide). DNA concentration and purity was monitored on 1% agarose gels, and an appropriate amount of sample DNA was diluted in centrifuge tubes. 16S rRNA/ITS genes of distinct regions (16S V4, ITS1) were amplified used specific primer with the barcode. PCR primers correspond 16S V4 region primers 515F (5′-GTGCCAGCMGCCGCGGTAA-3′) and 806R (5′-GGACTACHVGGGTWTCTAAT -3′) for identification of bacterial diversity; ITS1 primers (ITS5-1737F and ITS2-2043R) were used to identify fungal diversity.

### 2.6 Statistical analysis

The statistical analysis was performed using the IBM SPSS software package (version 24.0, SPSS Inc., Chicago, IL, USA) and R software environment (version 4.0.5, R Core Team, 2021). One-way analysis of variance (ANOVA) was performed using SPSS to test the significance of differences in soil physicochemical properties and P characteristics among stands of different ages. Significance levels were set at *P*< 0.05 and *P*< 0.01.

## 3 Results

### 3.1 Physicochemical indicators and nutrient characteristics of rhizosphere soil

Soil pH was significantly higher in stand A1 (pH = 4.58) than in other stands, and the differences between other stands were not significant (**Table 4**). As stand age increased, soil water content (WC) showed an increasing trend (except for A9) and was significantly highest at A42. Soil organic matter (SOM), total nitrogen (TN) and nitrate nitrogen (NN) showed an increasing and then decreasing trend, all reaching a maximum at A8. Soil total potassium (TK) was significantly (*P*< 0.05) highest at A42 and available potassium (AK) was higher at A9 than other stand ages but not significant. Ammonium nitrogen (AN) increased with stand age to a significant maximum at A2 and then continued to decline. In conclusion, the contents of WC, SOM, TN, AK and NN showed an increasing trend in the first eight years after afforestation.

**Table 4:** Physicochemical properties and nutrient content of rhizosphere soil.

	Stand age	Mean ± SD		Stand age	Mean ± SD
pH	A1	4.576 ± 0.356a	Total potassium,TK (g·kg^−1^)	A1	7.593 ± 1.598b
	A2	3.401 ± 0.105b		A2	4.383 ± 0.632b
	A3	3.494 ± 0.087b		A3	6.704 ± 1.528b
	A8	3.614 ± 0.108b		A8	6.917 ± 0.214b
	A9	3.474 ± 0.005b		A9	6.538 ± 1.025b
	A42	3.694 ± 0.16b		A42	14.531 ± 2.897a
Water content, WC (%)	A1	8.661 ± 0.24c	Ammonium nitrogen, AN (mg·kg^−1^)	A1	6.606 ± 0.633b
	A2	9.035 ± 0.703c		A2	11.079 ± 1.714a
	A3	9.472 ± 1.044c		A3	6.429 ± 1.194b
	A8	15.069 ± 1.451b		A8	5.064 ± 1.631bc
	A9	10.092 ± 0.036c		A9	4.74 ± 0.09bc
	A42	24.136 ± 1.335a		A42	3.114 ± 0.102d
Soil organic matter, SOM (g·kg^−1^)	A1	11.977 ± 0.092f	Nitrate nitrogen, NN (mg·kg^−1^)	A1	2.934 ± 0.055d
	A2	16.65 ± 0.488e		A2	3.559 ± 0.276d
	A3	27.969 ± 0.436c		A3	5.163 ± 0.204c
	A8	68.522 ± 1.408a		A8	13.583 ± 1.122a
	A9	48.272 ± 1.845b		A9	7.354 ± 0.271b
	A42	23.705 ± 0.227d		A42	6.43 ± 0.583b
Total nitrogen, TN (g·kg^−1^)	A1	1.373 ± 0.156c	Available potassium, AK (mg·kg^−1^)	A1	39.152 ± 1.277a
	A2	1.721 ± 0.365bc		A2	51.204 ± 2.638a
	A3	2.427 ± 0.428ab		A3	75.761 ± 3.773a
	A8	3.401 ± 0.384a		A8	76.626 ± 10.219a
	A9	3.271 ± 0.62a		A9	108.78 ± 0.765a
	A42	1.7 ± 0.3bc		A42	60.152 ± 7.385a

Different lowercase letters following the data indicate significant differences between stand ages at the level of 0.05.

### 3.2 Soil P availability and structure of P fractions

**Figure 1:**
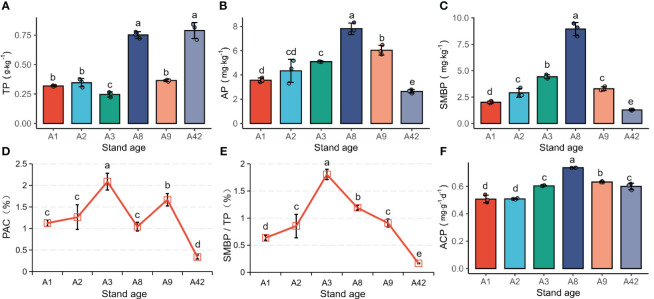
Characterisation of phosphorus factors of rhizosphere soil. TP, total phosphorus; AP, available phosphorus; SMBP, soil microbial biomass phosphorus; PAC, phosphorus activation coefficient; ACP, activity of acid phosphatase. Different lowercase on vertical bars indicate significant differences at the levels of 0.05.

Soil total phosphorus (TP) was significantly (*P*< 0.05) higher in A8 and A42 than in other stands, and the difference between other stands was not significant. Soil available phosphorus (AP), microbial biomass phosphorus (SMBP) and acid phosphatase activity (ACP) all increased significantly with stand age during the first eight years after afforestation, and then declined significantly, being lowest in the A42. Both phosphorus activation coefficient (PAC) and SMBP/TP were significantly highest at A3 and lowest at A42, with an overall trend of increasing and then decreasing (**Figure 1**).

**Figure 2:**
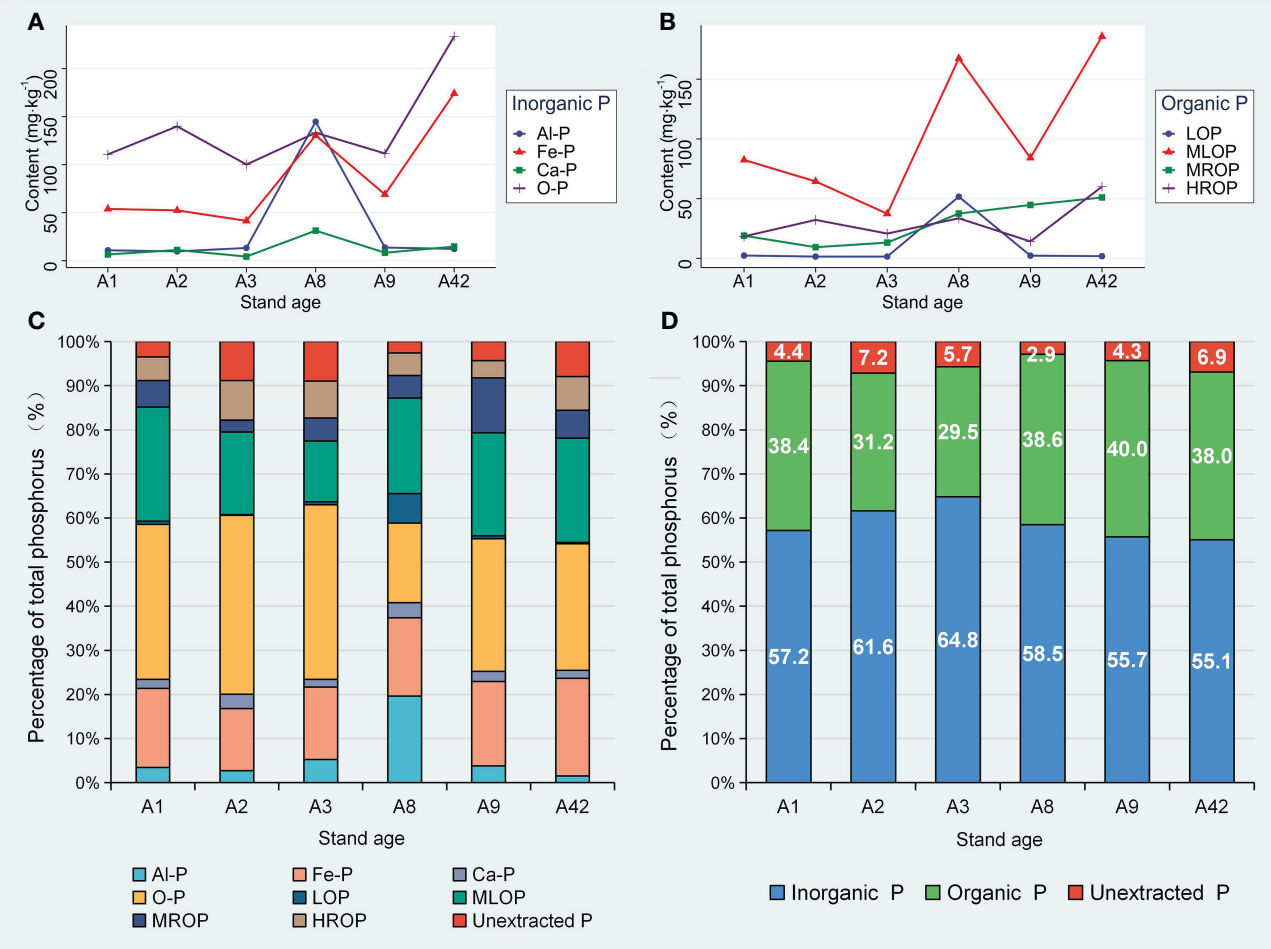
Changes in organic and inorganic phosphorus content of different forms in rhizosphere soils. Al-P: aluminium-bound phosphorus; Fe-P: iron-bound phosphorus; Ca-P: calcium-bound phosphorus; O-P: insoluble phosphorus (iron oxide gel-coated phosphate); LOP: easily mineralised and absorbed by plants; MLOP: relatively easy to mineralise and absorbed by plants; MROP: more difficult to mineralise and difficult to absorb by plants; HROP: more stable, insoluble and difficult to mineralized and basically not absorbed by plants; Organic P: numerically equal to the sum of the contents of the four organic phosphorus forms, Inorganic P: numerically equal to the sum of the four inorganic phosphorus forms; Unextracted P: numerically equal to the total phosphorus content minus total organic phosphorus and total inorganic phosphorus.

In all stands, the inorganic P (IP) fraction was highest in the form of O-P, followed by Fe-P (except for A8), while aluminum-bound P (Al-P) and calcium-bound P (Ca-P) were lower (**Figure 2A**). Moreover, among the organic P (OP) fractions, moderately labile organic P (MLOP) was consistently remained higher in all stands and reached its highest (185.8 mg·kg^-1^), at A42 followed by highly resistant organic P (HROP) and moderately resistant organic P (MROP) and the lowest levels of labile organic P (LOP), except at A8 (**Figure 2B**). The proportion of unextracted P to TP remained low throughout, less than 7.2% in all stands; the proportion of IP was always higher than that of OP, and with increasing stand age, the IP showed a trend of increasing and then decreasing, while OP projected an opposite trend (**Figure 2D**).

### 3.3 Correlation of phosphorus availability with other physicochemical indicators in rhizosphere soil

**Figure 3:**
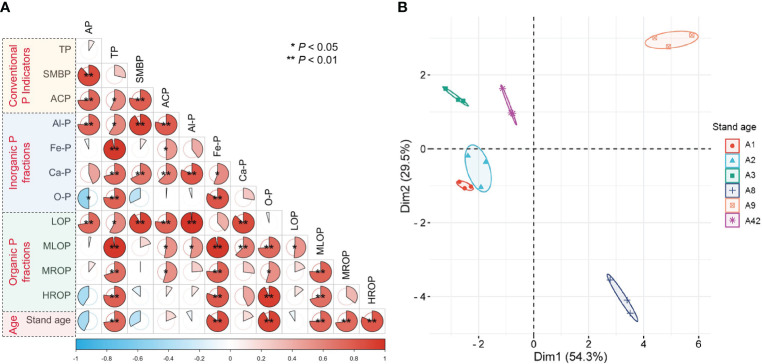
Correlation analysis **(A)** and principal component analysis **(B)** of changes in soil P indicators with stand age. The P indicators include TP, AP, SMBP, PAC, ACP, OP, IP, Al-P, Fe-P, Ca-P, O-P, LOP, MLOP, MROP and HROP, and they mean the same as the captions in **Figures 1** and **2**. Different colored ellipses in **(B)** represent different ages of stands, and the size of the ellipse indicates the 95% confidence interval.

The results of correlation analysis (**Figure 3A**) showed that AP was significantly (*P<* 0.01) positively correlated with SMBP, ACP, Al-P, and LOP; AP was significantly (*P<* 0.05) negatively correlated with O-P, and negatively correlated with HROP and Fe-P. TP was either significantly or highly significantly positively correlated with ACP, four inorganic P fractions, and four organic P fractions. In addition, SMBP and ACP were all highly significantly positively correlated with ACP, Al-P, and LOP. Importantly, stand age was positively correlated with TP, Fe-P, O-P, MLOP, MROP, and HROP; however, stand age was negatively, but not significantly, correlated with AP, SMBP, Al-P, and LOP.

The principal component analysis (**Figure 3B**) showed that axes 1 and 2 together explained 83.8% of the variation in the P characteristics for all stand ages. This shows that there are significant differences in P availability indexes and P fractions among different stand ages, especially between sample plots of A8 and A9 and those of other ages. The closer distance of sample points between sample plots A1, A2 and A3, means that the differences in rhizosphere P characteristics were small in the early years of afforestation; however, the differences in rhizosphere P properties gradually increase with the increase of stand age.

### 3.4 Comparative analysis of bacterial and fungal community composition and diversity in rhizosphere soil

**Figure 4:**
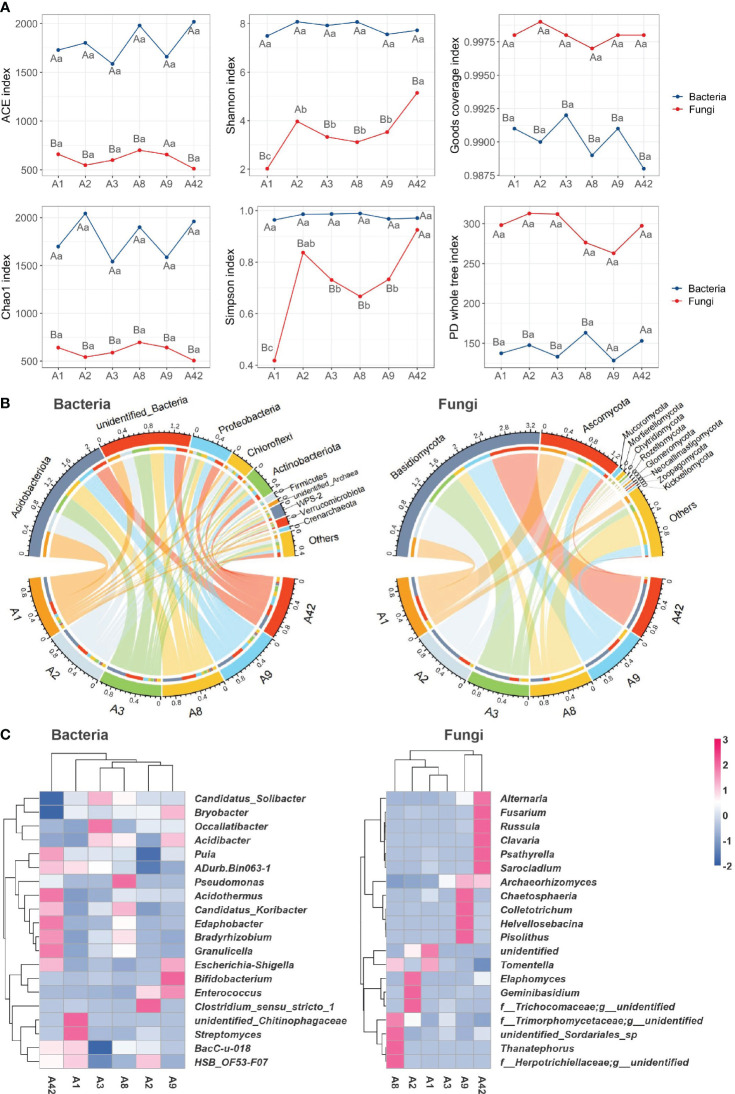
Microbial α diversity index **(A)**, Distribution of microbes in the top 10 phyla over stand ages **(B)** and Cluster analysis of microbes in the top 20 genera **(C)**. Different uppercase letters in **(A)** indicate significant differences in diversity indices between bacteria and fungi at the same stand age, and different lowercase letters indicate significant differences between different stand ages (*P* < 0.05). The clustering tree shows the similarity between samples, the shorter the branches between samples, the more similar the two samples are. The vertical branches in the clustering diagram represent different age groupings and the horizontal branches represent different genera. The colour gradient reflects the similarities and differences in the composition of multiple samples at the genus level.

The ACE and Chao1 index for bacteria were much higher than for fungi and varied synchronously with time, while Shannon and Simpson showed little variation with stand age. Shannon and Simpson for fungi were significantly lowest in the 1A stand and increased significantly in the 2nd year, reaching a maximum at 42 years of age. The Goods coverage index was greater than 0.996 for all the fungi in the stand, whereas it ranged from 0.9875 to 0.9925 for the bacteria, indicating that the fungi in this study were sequenced to a greater depth than the bacteria and that the affinities between the species comprising the fungal community were more complex (**Figure 4A**).

The compositional structure and diversity of dominant taxa of bacteria and fungi were analyzed at the phylum and genus levels, while characterizing the response of these key microorganisms to stand age. We found that the top 20 genera of the bacterial belonged to the phyla *Acidobacteriota*, *Actinobacteriota*, *Chloroflexi*, *Firmicutes*, *Proteobacteria*, and *Verrucomicrobiota*; the top 20 genera of fungi belonged to the phyla *Basidiomycota* and *Ascomycota*; and the microbial communities belonging to these phyla were strongly associated with all stand ages (**Figure 4B**), which shows that they are the key taxa in the dominant microbial communities. The clustering lines of the heat map (**Figure 4C**) show that, for both bacteria and fungi, the clustering distance between A42 and the other stand ages was farther, showing that the species compositions of the A42 stand differed more from those of the stands with shorter silvicultural time, and had the highest number of high-abundance genera, followed by the A9 stand. The distance of fungal community clustering was increasing with increasing stand age, and the larger the age span, the more distant they were from each other and the less similar their microbial compositions were.

### 3.5 Interactions of phosphorus factors and physicochemical indicators with microbial communities

**Figure 5:**
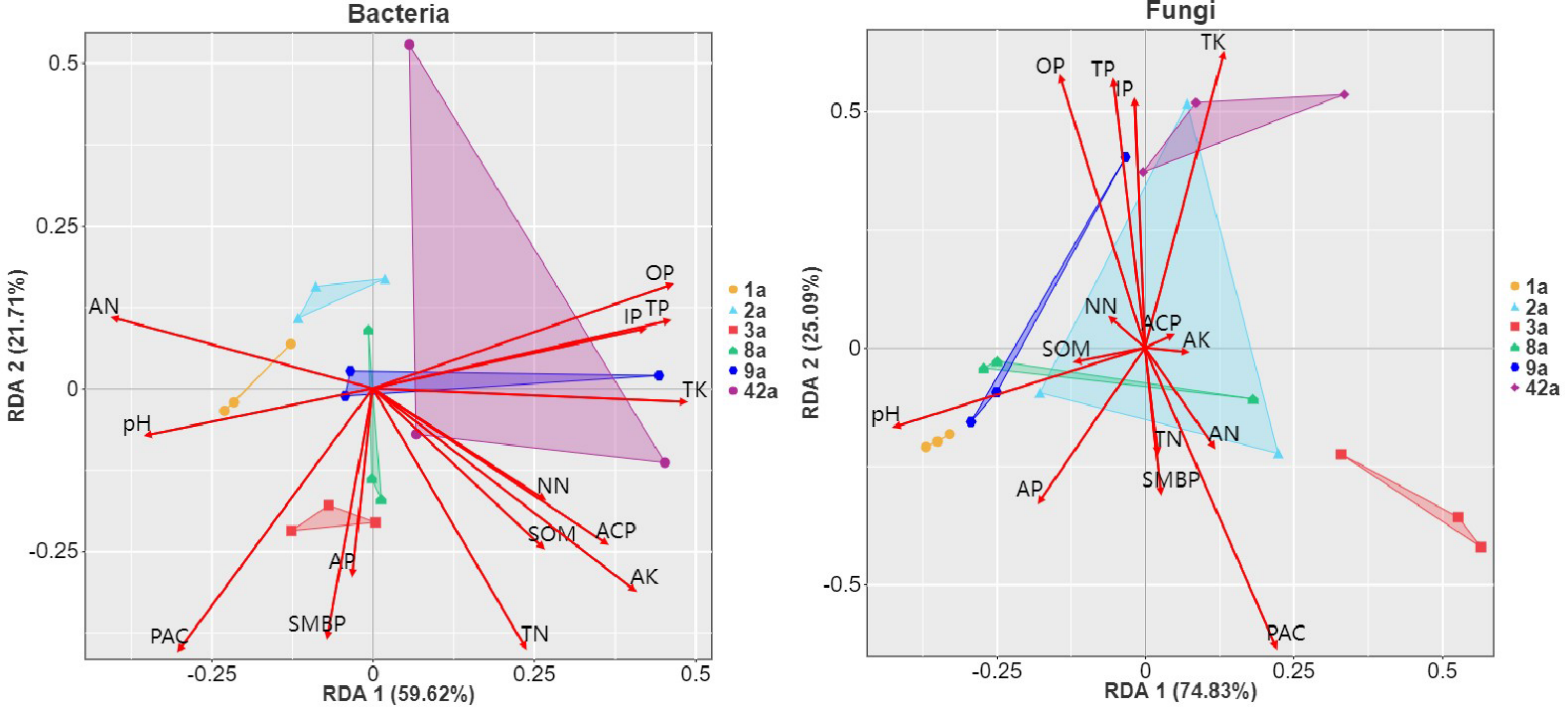
RDA analysis of microbial communities and physicochemical indicators. Triangle shading indicates microbial community diversity at different stand ages, and red line segments with arrows indicate different environmental factors. In the analysis of differences in bacterial community abundance, environmental factors were better at differentiating sample data on RDA axis 1 than axis 2, indicating that AN, pH, OP, TP, IP, TK, AK and ACP had a greater impact on bacterial community diversity; followed by PAC, SMBP and TN on axis 2. Among the effects on fungal community diversity, axis 1 contributed 74.83% of the variation in fungal community abundance. pH, AP and PAC were more correlated with axis 1 and they contributed more to microbial diversity, followed by environmental factors such as OP, TP, IP, TK and SMBP for axis 2.

The redundancy analysis (RDA) axes 1 and 2 together explain 81.33% of the variation in bacterial diversity with respect to environmental factors and 99.92% of the variation in fungi, suggesting that RDA analysis explains the effect of most environmental factors on species multiplicity (**Figure 5**). The results show that TP, OP, IP and ACP, potassium factors such as TK and AK, and nitrogen factors represented by AN were the main driving forces for changes in bacterial community structure, while pH, AP and PAC were also key drivers of variation in fungal community abundance over time. Factors centred on pH and PAC drive the development of rhizosphere microbial communities in *P. chinensis* plantations at long time scales.

**Figure 6:**
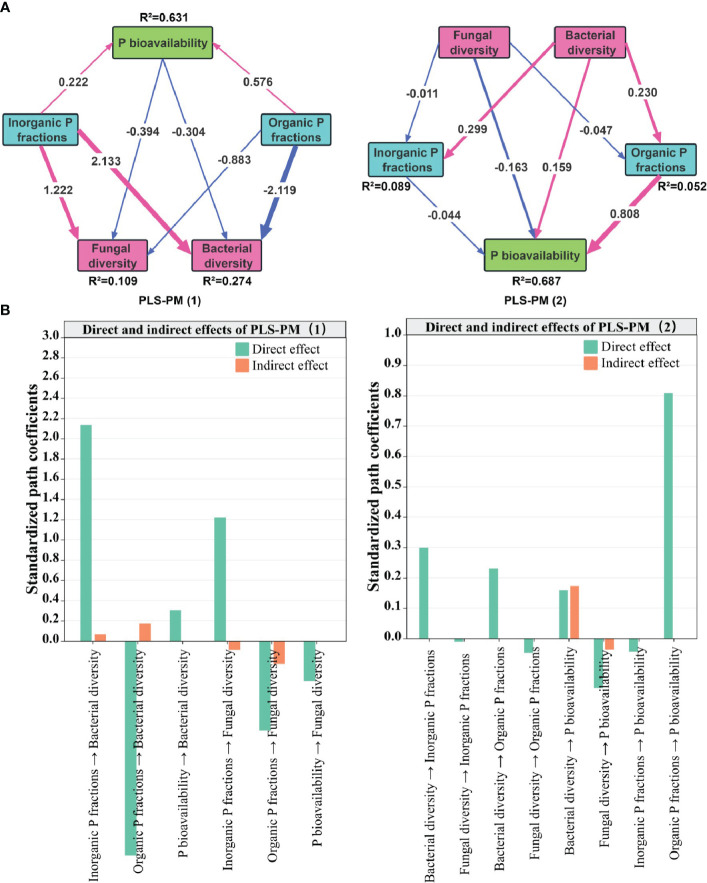
Directed graph of the partial least squares path model (PLS-PM) **(A)** and the standardized path coefficients for direct and indirect effects **(B)**. Note: the red and blue line segments represent positive and negative impacts, respectively, and the thickness of the line segment represents the magnitude of the impact.

The partial least squares path model (PLS-PM) results showed that for fungal and bacterial diversity, with R^2^ of 0.109 and 0.274, respectively, Inorganic P fractions showed a significant positive effect (**Figure 6A**), whereas both P bioavailability and Organic P fractions showed a negative effect, which was mainly dominated by direct effects (**Figure 6B**). In contrast, for P bioavailability, with an R^2^ of 0.687, fungal diversity and Inorganic P fractions exerted a negative effect, whereas Bacteria and Organic P fractions exerted a positive effect, with Bacteria having a greater indirect effect on P bioavailability. This shows that the microbial community had a greater effect on P availability than the effect of P factors on microbial diversity.

## 4 Discussion

### 4.1 Phosphorus availability, physicochemical properties and nutrient characteristics of rhizosphere soil

The pH of the rhizosphere soil of the stands at all ages in this study was acidic. With the increasing stand age, rhizosphere SOM, total N, available N, P, K, SMBP and ACP showed a trend of increasing and then decreasing (**Figure 1** and **Table 4**). It may be that the decrease in extracellular enzyme activity led to weaker SOM decomposition, thus reducing the amount of nutrient return (Wang et al., 2022). Root secretions from young trees usually cause nutrient enrichment, while increased nutrient demand by mature trees leads to a reduction in soil nutrients (Ma et al., 2007; Vitousek et al., 2010; Bell et al., 2014). AP content declined significantly from the 9th year after afforestation and was already below 3 mg/kg by 42 years, and other studies have shown a general scarcity of AP in mature forest soils in subtropical China (Wu et al., 2017; Xu H, et al., 2021). Among Dipterocarpaceae stands in the southeastern subtropical region, soil AP content is also reduced by the acidic environment, which limits the tree growth (Fujii et al., 2010; Fujii, 2014). We found that changes in SMBP and AP were synchronized, as rhizosphere soil microorganisms play a key role in mediating P activation (Moreau et al., 2019).

Both PAC and SMBP/TP first increased and then showed a decline, dropping to a minimum at 42 years. It may be related to the fact that acidic red soils have a strong P uptake capacity, and the slow-release properties of P fertilizer continue to exert fertilizing effects during the first 3 years after afforestation (Ngoze et al., 2008). Other evidence found that total N, as well as effective N, P, and K, were being lost from *Pinus yunnanensis* and *Pinus massoniana* at different stand ages (including young forest, medium forest, and near-mature forest) in the acidic red soil zone of southern China, and the rate of loss was faster than that of the natural forests in the control group (Selvalakshmi et al., 2018; Sun et al., 2023). Another study in *Pinus massoniana* plantations in southwestern China showed that the effectiveness and utilization of soil nutrients were higher in young stands than in old stands, and that the middle-age stand period was an important time point for influencing soil properties. Thus, in order to improve the effectiveness of soil nutrients and ensure the sustainable utilization of soil resources, it is necessary to increase the input of N and especially P (Yin et al., 2021). Therefore, it is necessary to keep the fertilizer for a longer period after the establishment of *P. chinensis*, which will be beneficial to maintain the high soil P availability and the number and activity of P-dissolving functional microorganisms. In general, low and medium density stands have significantly more favorable physicochemical properties and higher soil quality (Ali et al., 2019; Lei et al., 2019; Wang L, et al., 2023). However, although the silvicultural density of A42 stand was significantly lower than that of other stands in this study, its soil was significantly lowest in ammonium nitrogen, available phosphorus, microbial biomass phosphorus content and phosphorus activation coefficient. This indicates that after 42 years of afforestation, the effect of time span on soil nutrient effectiveness was sufficient to mask the effect of stand density.

The overall trend of increasing content of stable inorganic P (O-P, Fe-P) and organic P (MLOP, MROP) (**Figures 2A, B**) was probably due to the large amount of P in the strongly acidic soils being more readily bound to iron ions, leading to an increase in Fe-P and O-P content, while the more stable organic P also accumulated over time because it could not be mineralized by microorganisms. This leads to a severe limitation of P availability and SMBP as well as TN and AK could facilitate the conversion of TP to AP because soil microorganisms modulate P cycling processes (Marie and Meike, 2017). The decrease in the proportion of inorganic P to TP with increasing stand age was accompanied by an increase in the proportion of organic P (**Figures 2C, D**), because on the one hand, trees deplete the effective state of inorganic P in the soil, and on the other hand, the decrease in SMBP and ACP slows down the mineralization of organic P to available P (Taranto et al., 2000; Zhu et al., 2022). SOM with the available state of N and K elements could increase the AP content under the combined effect of acid phosphatase and microbial catabolism. The reason may be that organic matter and enzymatic activity are the main drivers regulating AP, and microbial metabolism enhances the amount of acid phosphatase to increase P availability (Lydia and Peter, 2000; Lu et al., 2022). We found an important effect of stand age on P effectiveness and transformation processes in rhizosphere soils, with significant divergence in P nutrient environments on a temporal scale (**Figure 3**), which is closely related to the driving role of microbial communities and secretion of acidic substances from the root system (Xu et al., 2022).

### 4.2 Characteristics of soil microbial communities in the rhizosphere at different ages

In *P. chinensis* plantations, rhizosphere soil bacteria maintained high and stable community abundance and evenness over long time scales, while fungal community composition varied considerably with age (**Figure 4**). Consistent with our findings, studies in *Ormosia hosiei* planted forests in southern China showed that forest age had no effect on bacterial diversity but significantly affected fungal community diversity (Wan et al., 2021). The PD whole tree index indicates that fungi are more phylogenetic than bacteria, have more complex affinities, and have more distinct clustering differences among different stand ages. The ACE and Chao1 indexes indicate that the total number of bacterial species far exceeds that of fungi, which is consistent with the microbiota characteristics of most forest soils (Wu et al., 2017). The ratio of fungal to bacterial populations rises with stand age, and certain fungi are symbiotic with plant roots for nutrient uptake and absorption, and therefore grow more favorably in nutrient-poor soils (Hu et al., 2022). The changes in Shannon and Simpson index are consistent with the findings of Lan et al (Lan et al., 2019). and their changes are mainly influenced by changes in silvicultural time.

This study showed that the longer the time after plantation, the more obvious differences in microbial community composition will be, especially the species composition of fungal communities will be richer, which is consistent with the results of soil studies in subtropical *Pinus elliottii* plantations (Wu et al., 2017). This is corroborated by other studies that soil microbial diversity in Guangxi is highly heterogeneous, and this heterogeneity is mainly determined by the stand age and soil nutrient effectiveness (Peng et al., 2021). Studies in *Pinus tabuliformis* plantations of different stand age, resulted in the abundance of most dominant bacterial and fungal communities being significantly correlated with organic carbon, total N, C:N ratio, available N, and available P, indicating the dependence of these microorganisms on soil nutrients (Dang et al., 2017). We found stronger correlations between bacterial and fungal communities and total N, total P, total K, and total organic P with increasing stand age. Therefore, in addition to external factors such as forest age that influence rhizosphere microorganisms, the interactions between soil nutrients and microbial communities within rhizosphere environments should be given sufficient attention, and our study has shown that the diversity of bacterial and fungal communities responds differently to soil nutrient content and its effectiveness.

### 4.3 Interrelationships between phosphorus availability, microorganisms and environmental factors

Soil microbial assembly was less environmentally constrained due to an increase in resource availability (Wang Y, et al., 2023). This has implications for our study, where we found that although soil nutrient resources decreased with stand development, the structural diversity of soil microbial communities in the rhizosphere soil of *P. chinensis* plantations did not decrease as a result, and even showed higher species richness and homogeneity in the later stages of silviculture. This suggests that the rhizosphere of *P. chinensis* may directly or indirectly shape the microbial community in favor of resisting the low P environment, a mechanism that has been reported in other studies (Chan et al., 2021). It is also possible that low P and P transformation exacerbate the interdependence between microbial communities and plant biomass and increase the abundance of microbial taxa with P mobilization capacity (Bulgarelli et al., 2022; DucoussoDétrez et al., 2022). Increasing forest age makes bacteria and fungi more specific and bacterial-fungal associations greater (Li et al., 2022). For example, bacteria of the Acidobacteria, Firmicutes and Proteobacteria phyla are significantly increased (Pereira et al., 2017). These rich microbial taxa were the main drivers in maintaining soil nutrient cycling and multifunctionality over time (Xu H, et al., 2021). The above evidence may corroborate our findings.

Rhizosphere soil microbial community composition was positively correlated with soil nutrient effectiveness (Xu M, et al., 2021). In our study, these types of bacteria were also in a dominant position, perhaps implying that P deficiency leads to the dominance of acidophilic and phosphate solubilizing bacteria in the rhizosphere soil of *P. chinensis*. The soils in this study area are P deficient in terms of AP and TP contents of rhizosphere soils (Bao, 2000). Microorganisms in such soils have a strong potential to mineralize organic P, and their activity and abundance profoundly affect the bioavailability of P (Liang et al., 2020; Tian et al., 2021). Even one study clearly pointed out that organophosphorus-solubilizing bacteria are the best species of P-solubilizing bacteria in red soil in southern China (Zhang et al., 2020). Additionally, fungi such as Basidiomycota, Ascomycota and Glomeromycota were also in dominant position. The mycelial network of these fungi could expand the uptake of nutrients such as P by plant roots to alleviate P limitation of the soil (Wang et al., 2022). Moreover, some studies have shown that trees become more dependent on these mycorrhizal fungi with increasing stand age (Rosling et al., 2016). In conclusion, we found that microbial diversity plays a more critical and dominant role in the relationship between P availability and microbial communities in rhizosphere soils, determining to some extent the direction of P fractions transformation and P bioavailability.

## 5 Conclusion

Rhizosphere soil of *Parashorea chinensis* plantation are strongly acidic, and soil P supply is severely limited by the adsorption of Fe^3+^ and high levels of stable ineffective state organic P in local soils. With increasing stand age, the mineralization of organic P to bioeffective inorganic P slowed down, the conversion of total P to available P weakened, and both soil organic matter and available state N, P, and K content decreased. Available P deficiency stimulates the growth and activity of bacteria of the phyla Acidobacteria, Firmicutes, and Proteobacteria, as well as fungi of the phyla Basidiomycota, Ascomycota, and Glomeromycota, rhizosphere microorganisms that play an important role in mitigating soil P limitation. In conclusion, we suggest that an appropriately extended fertilizer period would be beneficial in maintaining soil phosphorus supply capacity to sustain microbial ecological functions and tree growth.

## Data availability statement

The original contributions presented in the study are publicly available. This data can be found here: The National Center for Biotechnology Information https://www.ncbi.nlm.nih.gov/ accession number: PRJNA1091848.

## Author contributions

WL: Conceptualization, Software, Writing – original draft. SU: Methodology, Software, Writing – original draft. FL: Formal analysis, Writing – original draft. FD: Data curation, Writing – original draft. XH: Investigation, Supervision, Writing – original draft. SH: Investigation, Writing – original draft. YX: Resources, Supervision, Visualization, Writing – review & editing. MY: Funding acquisition, Project administration, Validation, Writing – review & editing.
